# A Rare Case of Pneumonia Caused by *Shewanella putrefaciens*

**DOI:** 10.1155/2012/597301

**Published:** 2012-10-22

**Authors:** Rajshree Patel, Albin Abraham, Johnson Thomas, Wanqing Zhi, Shadab Ahmed, Janice Verley

**Affiliations:** ^1^Department of Internal Medicine, Nassau University Medical Center, East Meadow, NY 11554, USA; ^2^Department of Infectious Disease, Nassau University Medical Center, East Meadow, NY 11554, USA

## Abstract

*Shewanella putrefaciens *is a gram-negative, nonfermentative, oxidase positive, motile bacillus that produces hydrogen sulphide. It is found widely in the nature especially in marine environments. In some very rare cases *Shewanella putrefaciens *can be a human pathogen. It can produce a wide variety of clinical syndromes including bacteremia as well as skin and soft tissue infections. However, pneumonia due to *S. putrefaciens *is rare; there are a total of 4 reported cases in the literature. We present a case of 63-year-old male who was presented to emergency room status after cardiac arrest, fell into sea water face down. On the second day of hospitalization, he was diagnosed to have pneumonia based on the clinical, radiological, and laboratory findings. Empirical antibiotic treatment with vancomycin and piperacillin/tazobactam combination was initiated. Gram-stained smear of endotracheal aspirate yielded gram-negative bacteria, and the isolate grown from endotracheal aspirate culture was identified as *S. putrefaciens *by Biomerieux API 20 NE technique. On review of the literature and according to culture and sensitivity results, therapy in our patient was changed to cefepime. Patient's pneumonia improved with treatment with cefepime. We believe that our patient developed pneumonia evidently caused by *S. putrefaciens*, after near drowning in sea water. The pneumonia resolved after treatment with cefepime.

## 1. Introduction

*Shewanella putrefaciens* is a gram-negative, nonfermentative, oxidase-positive, motile bacillus that produces hydrogen sulphide. It is found widely in the nature especially in marine environments. In some very rare cases *Shewanella putrefaciens *can be a human pathogen. It can produce a wide variety of clinical syndromes including bacteremia as well as skin and soft tissue infections [1]. It is most commonly thought of as a contaminant along with other bacteria or as a saprophyte, surviving with other organisms on previously damaged tissues in the body. However, pneumonia due to *S. putrefaciens *is rare; there are a total of 4 reported cases in the literature. Here we report a rare case of pneumonia caused by *Shewanella putrefaciens*.

## 2. Case Report

**Figure 1:**
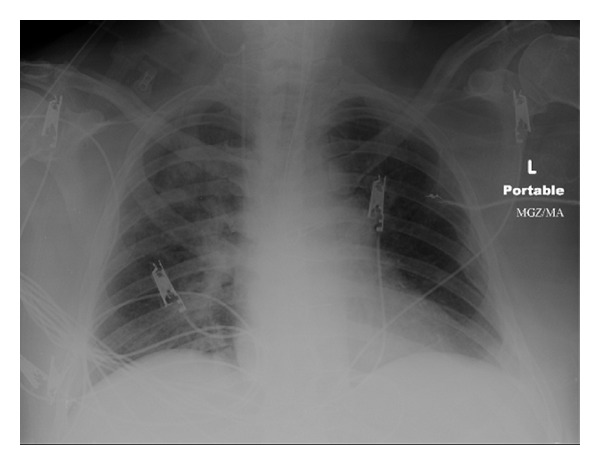


A 63-year-old Caucasian male was brought to the emergency room after having been found unresponsive. According to his family, he was at a local beach in Long Island, NY, waist-deep in the sea, when he was witnessed to have lost consciousness following which he fell face down into the water. He was resuscitated and intubated at the scene by emergency medical services. In the emergency room, he was diagnosed as having a NSTEMI and was admitted to the cardiac intensive care unit for further management. On the second hospital day, physical exam was significant for a low-grade temperature of 100.3 F and 102 F with bilateral coarse breath sounds and scattered rhonchi. Chest X-ray was as shown in Figure 1 showing developing infiltrates. The patient was empirically started on vancomycin and piperacillin/tazobactam at this point. Respiratory cultures from endotracheal aspirates were done the same day. He grew gram-negative bacteria which were later on the 3rd day identified as *Shewanella putrefaciens *by Biomerieux API 20 NE technique. Negative break point panel was performed and found organism being sensitive to aztreonam, cefepime, ceftazidime, ciprofloxacin, gentamicin, levofloxacin, meropenam, and piperacillin/tazobactam and resistant to imipenem. Blood cultures were done on a day when the patient spiked and 5 days after initial blood culture, all blood cultures were negative. In view of clinical and radiological signs and positive respiratory culture for *S. putrefaciens*, diagnosis of pneumonia secondary to *S. putrefaciens* was confirmed. On review of the literature and response of a previous reported case to cefepime, therapy in our patient was changed to cefepime IVPB 1 gm every 12 hours [3]. 24 hours following treatment the patients fever abated and he showed significant clinical improvement. He was later successfully extubated and has been doing well since. 

## 3. Microbiology

*Shewanella putrefaciens *known earlier as *Alteromonas putrefaciens *or *Pseudomonas putrefaciens *is the only nonfermentative gram-negative rod that produces hydrogen sulfide [2]. *S. putrefaciens *and *S. algae *infection correlates with the temperature and salinity of seawater. This means *Shewanella *infections occur in warm climates or during warm summers in temperate climates. In contrast to *S. putrefaciens*, *S. algae *produces mucoid colonies with beta-hemolysis on sheep blood agar, grows at 42 degrees C and in NaCl 6%, and reduces nitrite [5]. However *S. algae *cannot produce acid from maltose. Important differential characteristics between the two species include the ability of *S. algae *to produce mucoid colonies with beta-hemolysis on sheep blood agar, to grow at 42 degrees C and in NaCl 6% w/v, and to reduce nitrite and an inability to produce acid from maltose, all in contrast to the characteristics of *S. putrefaciens* [6]. As the two species seem to have different pathogenic potential for humans, correct identification is important, and this is possible in routine clinical microbiology laboratories.

## 4. Discussion

The infection from *S. putrefaciens *most commonly involves skin and soft tissue associated with damage to skin (trauma, cut, ulcer) and otitis media. Primary bacteremia with fulminant course is also seen in immunocompromised patients. *S. putrefaciens *does not commonly cause lower respiratory tract infection. Respiratory colonization with the possibility of infection has been identified in rare cases from isolates cultured from sputum and pleura and transthoracic needle aspiration cultures [2]. It is likely that *S. putrefaciens *was introduced into our patient's respiratory tract from the seawater when he fell face down into it. Pneumonia associated with *S. putrefaciens *has been reported in 4 other cases. In one case the patient fell in a river during a watercraft accident and developed pneumonia due to *S. putrefaciens*, while in another case the patient developed respiratory colonization after near drowning in sea water [1–4]. The source of contamination with *S. putrefaciens *in the other two cases could not be confirmed.  Table 1 shows the summary of the 4 previously reported cases in addition to the case currently being reported [1–3, 6, 7]. The association between a positive culture and the clinical symptoms, with the improvement in clinical picture after the initiation of treatment leaves, no doubt about pathogenic character of the isolate in this patient. 

## 5. Conclusion

The patient developed pneumonia evidently caused by *S. putrefaciens*, after near drowning in sea water. The pneumonia resolved after treatment with cefepime.

## Figures and Tables

**Table 1:** Summary of the reported cases of pneumonia caused by *S*. *putrefaciens.*

Case	Age	Sex	Possible source	Diagnosed after days of hospitalization	Treatment outcome	Reference
1	64	M	?	38	Improved	[2]
2	39	M	River water	14	Improved	[3]
3	43	F	?	1	Improved	[1]
4	63	M	Sea water	2	Improved	This case
5	56	M	Sea water	3	Improved	[4]
